# Access to mass rapid transit in OECD urban areas

**DOI:** 10.1038/s41597-020-00639-3

**Published:** 2020-09-08

**Authors:** Vincent Verbavatz, Marc Barthelemy

**Affiliations:** 1 Université Paris-Saclay, CNRS, CEA, Institut de physique théorique, 91191 Gif-sur-Yvette, France; 2 École des Ponts ParisTech, Champs-sur-Marne, France; 3 Centre d’Etude et de Mathématique Sociales, CNRS/EHESS, 54 Boulevard Raspail, 75006 Paris, France

**Keywords:** Geography, Interdisciplinary studies

## Abstract

As mitigating car traffic in cities has become paramount to abate climate change effects, fostering public transport in cities appears ever-more appealing. A key ingredient in that purpose is easy access to mass rapid transit (MRT) systems. So far, we have however few empirical estimates of the coverage of MRT in urban areas, computed as the share of people living in MRT catchment areas, say for instance within walking distance. In this work, we clarify a universal definition of such a metrics - People Near Transit (PNT) - and present measures of this quantity for 85 urban areas in OECD countries – the largest dataset of such a quantity so far. By suggesting a standardized protocol, we make our dataset sound and expandable to other countries and cities in the world, which grounds our work into solid basis for multiple reuses in transport, environmental or economic studies.

## Abstract (summary)

**Table:** 

Measurement(s)	people near transit • access to mass rapid transit
Technology Type(s)	digital curation • computational modeling technique
Factor Type(s)	OECD country
Sample Characteristic - Environment	city
Sample Characteristic - Location	Canada • Australia • Kingdom of Spain • Portuguese Republic • Baltic states • Austria • Poland • Czech Republic • Hungary • United Kingdom • contiguous United States of America • French Republic • Italy • Germany • Greece • Scandinavia • Benelux • Mexico • South America

Machine-accessible metadata file describing the reported data: 10.6084/m9.figshare.12770162

## Background & Summary

Motorized transport currently accounts for more than 15% of world greenhouse gas emissions^1^. As most humans live in urban areas and two-thirds of world population will live in cities by 2050^2^, mitigating car traffic in cities has become crucial for limiting climate change effects^3–6^. Daily commuting is the main driver for passenger car use - about 75% of American commuters drive everyday (U.S. Department of Transportation, Bureau of Transportation Statistics, National Transportation Statistics. Table 1–41 at http://www.bts.gov (2016)) - while alternative transport modes such as public transportation networks are unevenly developed among countries and cities (List of Metro Systems, *Wikimedia Foundation* https://en.wikipedia.org/wiki/List_of_metro_systems, 2020).

Over the last decades, various attempts to assess the environmental impact of car use in cities have emerged from multiple fields, ranging from econometric studies to physics or urban studies^7–11^. A seminal result of transport theory, by Newman and Kenworthy^10^, correlated transport-related emissions with a determinant spatial criterion: urban density. Alternatively, Duranton and Turner^11^ claimed that public transport services were unsuccessful in reducing traffic, as transit riders lured off the roads are replaced by new drivers on the released roads. Such results, however, crucially lack both theoretical and empirical foundations^12–15^ and new research^16^ shows that the two main critical factors that control car traffic in cities are urban sprawl and access to mass rapid transit (MRT).

More generally, understanding mobility in urban areas is fundamental, not only for transport planning, but also for understanding many processes in cities, such as congestion problems, or epidemic spread^17,18^ for example. But what is a good measure of access to transit? Studies have mainly focused on the number of lines or stops^19–21^, length of the network or graph analysis^22–24^. Few works^16,25,26^, however, have considered investigating catchment areas of MRT stations, i.e. looking at the share of population living close to MRT stations, for instance within walking distance. Such conditions have however proved to be essential in explaining commuting behaviours and mobility patterns^16^.

The most detailed definition of such catchment metrics is the People Near Transit (PNT), and originates from a 2016 publication from the Institute for Transportation and Development Policy (IDTP)^25^. It produces a rigorous dataset of the share of population living close to transit (less than 1 km) for 25 cities in the world (12 in OECD countries). However, definitions of urban areas and rapid transit systems in that dataset are multiple and need to be refined while the number of cities must be expanded.

Hence, in order to expand our global knowledge of urban mobility, we need a common, unified and universal definition of access to public transit as well as sound measures of such a quantity. In this paper, we clarify its definition and propose what is to our knowledge the largest global dataset of PNT.

Our analysis uses functional urban areas (FUA) in OECD countries, a consistent definition of cities across several countries^27^. We restrict our measures to mass rapid transit, usually referring to high-capacity heavy rail public transport, to which we added light rails and trams. In our sense, mass rapid transit thus encompasses:

- Tram, streetcar or light rail services.
- Subway, Metro or any underground service.
- Suburban rail services.

Buses are not comprised in that definition. In contrast with^25^, we do not exclude any form of commuting trains based on station spacing or schedule criteria. As we detail it in the Method section, we identify services and corresponding stops with the General Transit Feed Specification (GTFS), a common format for public transportation schedules and associated geographic information (GTFS Static Overview. https://developers.google.com/transit/gtfs, 2020).

**Fig. 1:**
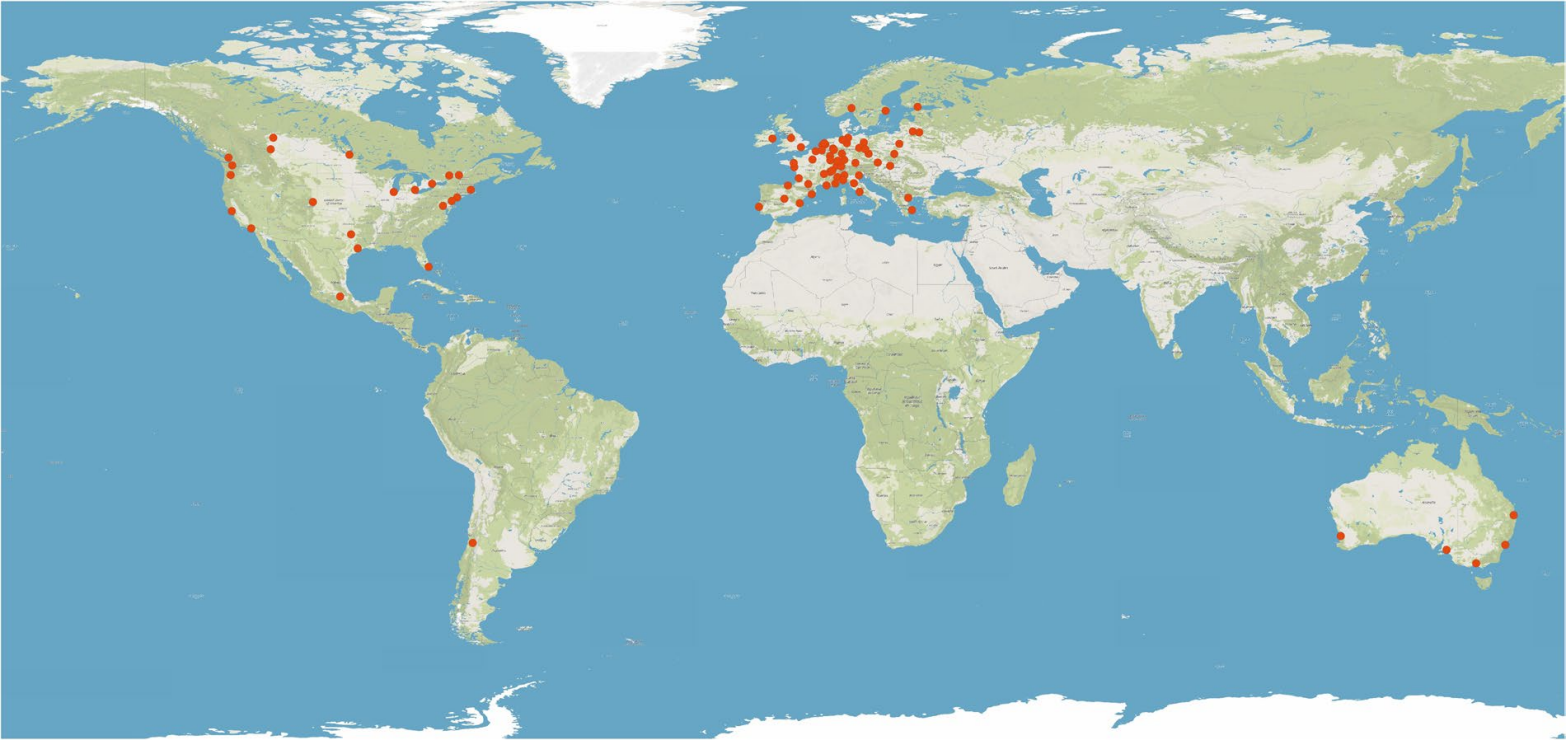
The 85 OECD cities for which we found data are mostly found in Europe and in North America (MapTiler Basic and MapTiler Topo. https://www.maptiler.com, 2020).

Crossing open-access information from public transport agencies in OECD urban areas with population-grid estimates of world population^28^, we publish here a list of 85 OECD cities (see Fig. 1) for which we were able to compute the People Near Transit (PNT) levels defined as the share of urban population living at geometric distances of 500 m, 1,000 m and 1,500 m from any MRT station in the agglomeration:

1 $${\rm{PNT(}}d{\rm{)}}=\frac{{\rm{population}}\,{\rm{s}}{\rm{.}}\,{\rm{t}}{\rm{.}}\,{\rm{euclidean}}\,{\rm{minimum}}\,{\rm{distance}}\,{\rm{ < }}\,d}{{\rm{total}}\,{\rm{population}}}$$

where *d* = 500, 1000, 1500.

We display on Tables 1 and 2 the 5 cities with easiest access to MRT (largest PNT) and the 5 cities with scarcest access to MRT (smallest PNT).

**Table 1:** Population Near Transit values: Share of population living within catchment area from a MRT station at thresholds 500 m, 1000 m and 1500 m. Top 5 cities with easiest (1000 m) access to MRT.

City	Country	Population	500 m PNT (%)	1000 m PNT (%)	1500 m PNT (%)
Basel	Switzerland	528811	57.78	80.15	86.96
Bilbao	Spain	986042	56.84	76.79	83.52
Geneva	Switzerland	592893	50.44	74.68	85.07
London	United Kingdom	11754700	43.09	72.56	85.8
Zurich	Switzerland	1329898	42.7	68.18	82.09

**Table 2:** Population Near Transit values: Share of population living within catchment area from a MRT station at thresholds 500 m, 1000 m and 1500 m. 5 cities with poorest (1000 m) access to MRT.

City	Country	Population	500 m PNT (%)	1000 m PNT (%)	1500 m PNT (%)
Winnipeg	Canada	846133	0	0	0
Detroit	United States	4263202	0.1	0.18	0.31
Houston	United States	6706227	0.98	2.28	3.54
Miami	United Sates	5964846	1.26	3.51	5.65
Dallas	United States	7294931	1.18	4.05	7.64

**Fig. 2:**
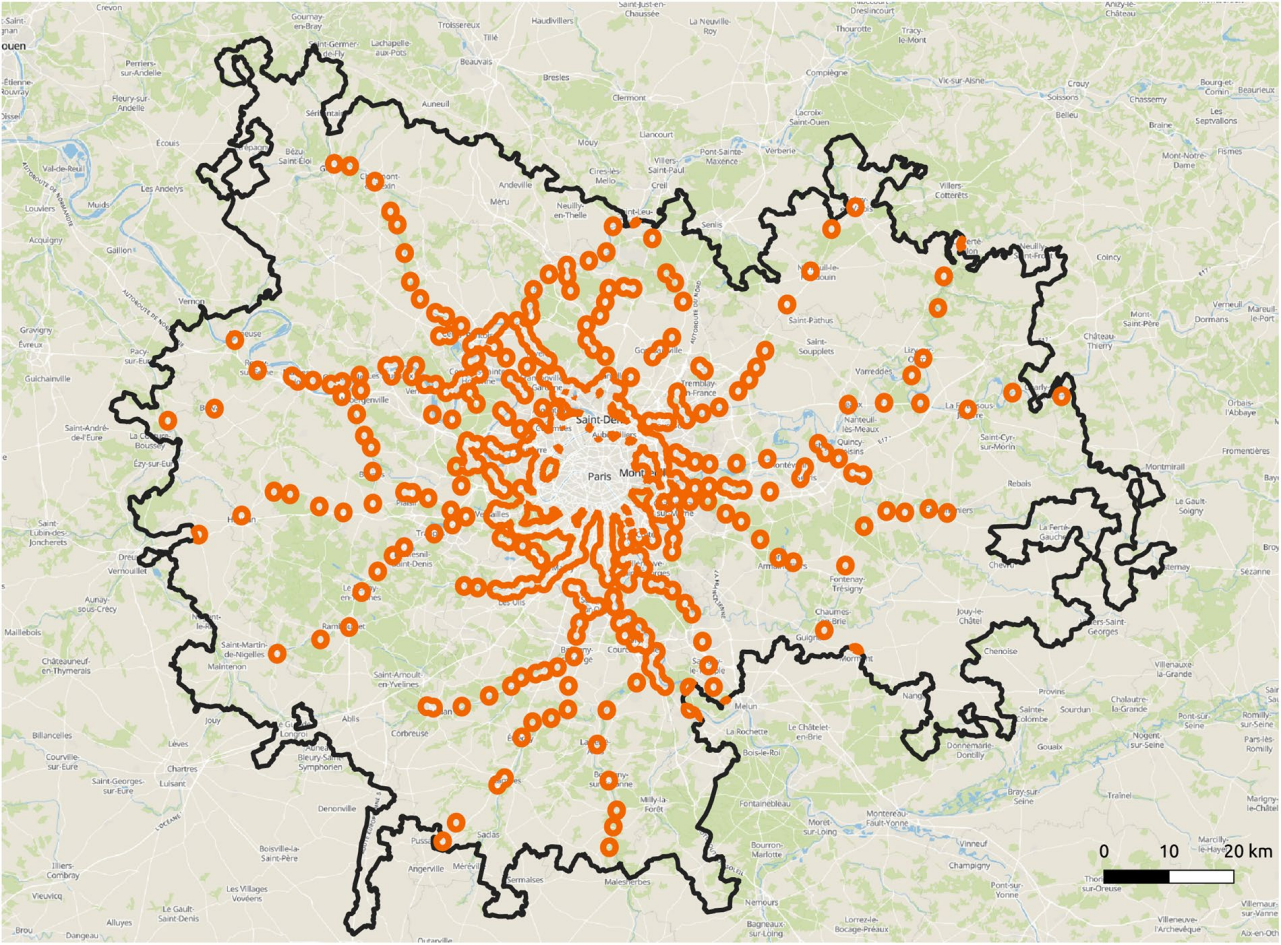
1000 m catchment areas of MRT stations (in orange) in Paris functional urban area (boundaries are in black) (MapTiler Basic and MapTiler Topo. https://www.maptiler.com, 2020).

We also provide for each city the population grid-maps with corresponding MRT access level, i.e. grid-maps of MRT catchment areas at different distances with population in each grid. As an example, Fig. 2 shows the 1000 m catchment area of MRT stations in Paris.

## Methods

### Residential populations for FUA

Our analysis relies on the 2015 residential population estimates mapped into the global Human Settlement Population (GHS-POP) project^28^. This spatial raster dataset depicts the distribution of population expressed as the number of individuals per cell on a grid of cells 250 m long. Residential population estimates for the target year 2015 are provided by CIESIN GPWv4.10^29^ and were disaggregated from census or administrative units to grid cells.

We downloaded population tiles that cover land on the globe in the Mollweide projection (EPSG:54009) and in raster format (.tif files). These raster data are made of pixels of width 250 m with associated value the number of people living in the cell. We processed the downloaded tiles with **Python** 3.7.6 (Python Language Reference, version 3.7.6 available at http://www.python.org) and package **gdal** 3.0.2 (GDAL/OGR, Geospatial Data Abstraction software Library, *Open Source Geospatial Foundation* https://gdal.org) to convert the raster files into vectorized shapefiles. The resulting shapefiles are comprised of polygons with field value the population in each polygon. Since the polygonization process merges adjacent pixels with common value into single polygons, populations for each polygon must be recomputed from polygon area and density through the simple following rule:

2 $${{\rm{Pop}}}_{polygon}={{\rm{Pop}}}_{pixel}\times \frac{{{\rm{Area}}}_{polygon}}{{{\rm{Area}}}_{pixel}}$$

where Area_*pixel*_ = 250 × 250 = 62500 m^2^. This leaves us with a list of 224 shapefiles of population that cover land area on earth.

By intersecting the resulting shapefiles with OECD shapefiles delineating Functional Urban Areas (FUA) in OECD countries^27^ (reprojected into Mollweide projection), we can build a population-grided dataset of cities in OECD countries.

These resulting files are the population substrates used for measuring population living close to MRT stations.

### Extracts of MRT stations from GTFS files

A common and *de facto* standard format for public transportation schedules and associated geographic information is the General Transit Feed Specification (GTFS Static Overview. https://developers.google.com/transit/gtfs, 2020).

A GTFS feed is a collection of at least six CSV files (with extension.txt) contained within a.zip file. It encompasses general information about transit agencies and routes in the network, schedule information such as trips and stop times and geographic information for stops (geographic coordinates).

The three main objects we require are:

- Routes: distinct routes in the network of a certain type. A route is a (one-direction) regular line, for instance a metro or bus line. The route types we use are (https://developers.google.com/transit/gtfs):

- Tram, Streetcar, Light rail. Any light rail or street level system within a metropolitan area.

- Subway, Metro. Any underground rail system within a metropolitan area.

- Rail. Used for intercity or long-distance travel.

- Cable tram. Used for street-level rail cars where the cable runs beneath the vehicle, e.g., cable car in San Francisco.

Our definition of MRT excludes bus and ferry types:

- Bus. Used for short- and long-distance bus routes.

- Ferry. Used for short- and long-distance boat service.

- Trips: trips are associated to a route and define a particular and scheduled trip between specific stations. For instance, the first train of the day is a trip.
- Stops: stops are geographic locations of the stops, stations and their amenities within the transit system. Stops are organized into a parent station and their amenities (e.g. platforms or exits).

Joining in this order the four tables **routes.txt**, **trips.txt**, **stop_times.txt** and **stops.txt** allows us to bind stops with their associated route types. We can thus discriminate between bus stops and metro stops and thereby select objects according to our definition of MRT.

In a nutshell each GTFS file can be processed to produce localized and route-typed stops.

### Measure of People Near Transit (PNT)

In order to measure PNT within urban areas, we must bind transit systems with their respective FUAs. We need to retrieve - and merge - all available GTFS files pertaining to a specific urban area and make sure that no rapid transit agency is excluded in the process.

Most GTFS files for cities in the world are collected by the OpenMobilityData platform (https://transitfeeds.com, 2020). For each city in our dataset, we cross-checked the OpenMobilityData with Wikipedia local network information (List of Metro Systems, https://en.wikipedia.org/wiki/List_of_metro_systems) to ensure that we considered all agencies of rapid transit within the urban area.

For some European countries (Germany, France), GTFS files were not availaible on OpenMobilityData and had to be retrieved from other sources (*GTFS für Deutschland* https://gtfs.de/ and *Open platform for French public data* https://www.data.gouv.fr). We also note that GTFS format is not common in South Korea, Japan and in the United Kingdom where we only found GTFS data for Manchester area on OpenMobilityData (https://transitfeeds.com) while we directly used station coordinates for London (*Transport for London*. TFL Station Locations available at https://data.london.gov.uk/dataset/tfl-station-locations).

We were thus left with a list of 85 urban areas in the world for which we had complete, reliable and extensive data. From route-typed stop coordinates within that dataset, we can extract MRT stops (excluding buses and ferries) and buffer - still using gdal - catchment areas for several distance thresholds: 500 m, 1000 m and 1500 m. Intersecting the resulting buffers with the population-grided shapefiles gives us the total population living within catchment areas, that can be expressed as a share of the total urban area population resulting in the value of the PNT metric. Our results are shown in Online-only Table 1.

## Data Records

The Data Record of PNT in OECD urban areas is available online on *Figshare*^30^.

PNT levels at distance thresholds: 500 m, 1 000 m and 1 500 m for the 85 Functional Urban Areas are shown on the Online-only Table 1. The list of transit agencies for each city is online along with PNT statistics (**mrt_access.csv**)^30^.

We also provide, for each city, grid-maps of population at different distances from MRT(**pops_close_to_MRT.zip**)^30^.

The Tables read as follows: Basel urban area has 528811 inhabitants, of which 57.78% live within 500 m of a MRT station, 80.15% within 1000 m and 86.96% within 1500 m.

## Technical Validation

The most thorough and exhaustive measure of PNT in urban areas in existing literature is a 2016 report from the Institute for Transportation and Development Policy^25^. To validate our results and our methodology, we compared them with those results.

Out of the 12 OECD cities considered in^25^, 11 are in our dataset: 5 in the United States, 2 in Spain, 1 in Canada, 1 in France, 1 in the United Kingdom and 1 in the Netherlands (see Table 3). Unfortunately, we found no data in the remaining city: Seoul.

**Table 3:** Comparison of MRT Share from the IDTP report^25^ with our estimations for 11 OECD cities. Discrepancies at first glance can be explained by different delineations of cities or transit systems. Applied on the same entities, results are similar.

City	Country	Population in^25^	Types in^25^	PNT Share (%) in^25^	Our PNT Share (%)	Our PNT Share (%) with^25^ criteria	Comments about^25^
Barcelona	Spain	3200000	Metro + LRT	76	65	74	Urban core and not FUA 1–suburban trains are *de facto* included in^25^
Boston	US	4650000	Metro + LRT	15	31	17	Excludes suburban trains
Chicago	US	9500000	Metro	14	13	13	/
London	UK	10000000	Metro + LRT + Suburban Rail	61	73	/	Some suburban trains are excluded in^25^
Los Angeles	US	13000000	Metro + LRT + BRT	11	9	/	We exclude Bus Rapid Transit
Madrid	Spain	5500000	Metro + LRT	76	60	72	Urban core and not FUA 1–suburban trains are *de facto* included in^25^
New York	US	19800000	Metro + LRT	35	45	34	Excludes suburban trains
Paris	France	12000000	Metro + Tram + Suburban Rail	50	63	/	Some tramlines are excluded in^25^
Rotterdam	Netherlands	1200000	Metro + LRT	55	41	50	Urban core and not FUA 1–suburban trains are *de facto* included in^25^
Vancouver	Canada	2300000	Metro	19	23	23	/
Washington	US	5800000	Metro	12	8	12	Urban core and not FUA

Out of these 11 cities, we had at first glance similar results for only two cities: Chicago (13% for both) and Vancouver (19% vs 23%). The discrepancies observed for the other cases stem from different definitions of cities and from the different transit systems that were taken into account. While we work with Functional Urban Areas (FUA) only, the authors of ^25^ mix two different definitions of cities: FUA and urban cores. By applying our method to urban cores and not functional urban areas, we found the same or similar results for Barcelona, Madrid, Rotterdam and Washington (see Table 3).

Also, the authors of ^25^ considered a definition of the LRT (Light Rail Transit) and Suburban Rail that depends on station spacing and schedule criteria. We didn’t choose this definition and for Boston and New York, we had therefore to exclude suburban trains - while keeping the definition of FUA - in order to retrieve results similar to those of Table 3. In contrast, the study^25^ took into account the Bus Rapid Transit for Los Angeles, that we decided to exclude. Finally, in Paris the authors of ^25^ considered that the so-called RER trains were comprised in Suburban Rail, but not Transilien trains, while we included both systems in our analysis.

The conclusion here is that for similar definitions for cities and transit systems, we obtain similar results, validating our method and calculations. In order to facilitate the comparison across future studies, we would recommend using the definition of cities given by Functional Urban Areas since it is very commonly used and already unified for OECD countries. Concerning transit systems, we think that it is more relevant and also verifiable to consider transit systems based on their types (Rail versus Road) rather that on spacing and schedule criteria that are specious and less universal. Hence, in comparing our results with results from the IDTP report^25^ and after checking on Table 3 that our methodology is correct, we decided to keep our unmodified estimations for the considered cities, despite the discrepancies with^25^.

For other cities in the dataset we have unfortunately found no existing data to compare with. Thus, we hope for future research to test and expand our estimations and results.

## Usage Notes

Easy code and hints are given on *Gitlab* (https://gitlab.iscpif.fr/vverbavatz/mrt-access-project).

We strongly recommand using GDAL (GDAL/OGR, Geospatial Data Abstraction software Library, *Open Source Geospatial Foundation* https://gdal.org, 2020) to handle geographic data with Python.

## Data Availability

Detailed code generating the database can be accessed from the source code hosted via *Gitlab* (https://gitlab.iscpif.fr/vverbavatz/mrt-access-project).

## Online-only Table

**Online-only Table 1:** Population Near Transit values: Share of population living within catchment area from a MRT station at thresholds 500~m, 1~000~m and 1~500~m for 85 OECD Functional Urban Areas.

City	Country	Population	500m	1000m	1500m
Adelaide	Australia	1368481	11.43	27.9	40.79
Amsterdam	Netherlands	2766282	21.4	39.14	52.66
Athens	Greece	3667934	41.73	63.26	74.43
Barcelona	Spain	4838161	41.65	65.24	77.82
Basel	Switzerland	528811	57.78	80.15	86.96
Berlin	Germany	4953645	38.39	63.02	76.13
Bilbao	Spain	986042	56.84	76.79	83.52
Bordeaux	France	1176238	21.94	41.5	53.34
Boston	United States	4167892	13.4	30.69	44.69
Bremen	Germany	1253514	21.64	38.37	50.62
Brisbane	Australia	2307430	9.27	25.63	37.82
Brussels	Belgium	2632048	34.35	43.43	46.6
Budapest	Hungary	2972657	34.3	48.55	55.79
Calgary	Canada	1492971	5.7	18.93	31.15
Chicago	United States	9608320	5.91	13.34	18.45
Cologne	Germany	1960557	30.43	55.07	68.99
Cracow	Poland	1392519	22.36	34.41	41.95
Dallas	United States	7294931	1.18	4.05	7.64
Denver	United States	2738183	3.03	9.53	17.91
Detroit	United States	4263202	0.1	0.18	0.31
Dresden	Germany	1317454	35.47	56.14	66.95
Dublin	Ireland	1866112	15.96	35.23	50.17
Dusseldorf	Germany	1541332	33.01	55.86	69.01
Edmonton	Canada	1324949	3.6	10.8	17.26
Florence	Italy	770710	15.22	25.25	31.03
Frankfurt am Main	Germany	2579579	28.35	56.11	72.33
Geneva	Switzerland	592893	50.44	74.68	85.07
Genoa	Italy	699462	11.3	26.02	36.9
Hamburg	Germany	3191585	16.6	39.5	55.71
Hanover	Germany	1272611	30.89	54.79	65.58
Helsinki	Finland	1451912	24.39	45.22	56.88
Houston	United States	6706227	0.98	2.28	3.54
Kaunas	Lithuania	380048	37.79	51.5	58.29
Lausanne	Switzerland	410089	32.63	65.0	80.17
Leipzig	Germany	972864	42.76	60.57	69.03
Lille	France	1360801	26.07	48.25	61.46
Lisbon	Portugal	2831367	18.17	41.66	55.96
London	United Kingdom	11754700	43.09	72.56	85.80
Los Angeles	United States	17712325	2.26	8.56	15.91
Luxembourg	Luxembourg	577309	16.1	39.06	55.64
Lyon	France	1963944	29.56	50.85	63.87
Madrid	Spain	6615767	33.94	59.62	70.89
Manchester	United Kingdom	3298781	16.34	43.57	64.61
Marseille	France	1779703	17.58	31.23	42.23
Melbourne	Australia	4466894	23.27	40.75	54.65
Mexico City	Mexico	20578866	8.8	20.6	28.89
Miami	United Sates	5964846	1.26	3.51	5.65
Milan	Italy	4966888	28.26	48.99	63.0
Montreal	Canada	4478991	10.88	25.82	37.75
Munich	Germany	2825789	36.74	62.44	75.31
Nancy	France	477056	16.42	36.16	52.43
Nantes	France	908423	21.41	40.08	50.82
New York	United States	19694439	27.02	45.35	56.68
Nice	France	848591	20.2	39.87	53.29
Oslo	Norway	1332133	30.16	47.59	53.67
Ottawa	Canada	1500455	2.18	7.46	12.03
Paris	France	12012223	37.16	62.7	77.56
Perth	Australia	1930198	4.88	15.66	27.01
Philadelphia	United States	6432106	4.55	14.34	23.81
Portland	United States	2262652	6.64	15.61	24.75
Prague	Czech Republic	2251032	35.21	59.46	73.52
Rennes	France	720142	10.18	23.61	35.69
Rome	Italy	4161006	25.93	46.97	58.33
Rotterdam	Netherlands	1823101	20.8	41.13	56.04
San Francisco	United States	6273368	10.92	25.15	37.74
Santiago	Chile	7182609	13.0	34.01	49.49
Seattle	United States	3620117	2.83	6.45	10.12
Stockholm	Sweden	2221640	34.96	60.65	72.73
Strasbourg	France	779704	27.56	51.27	63.81
Stuttgart	Germany	2662983	27.57	51.06	64.64
Sydney	Australia	4903571	13.91	34.98	50.88
Thessaloniki	Greece	1076231	1.38	5.62	11.54
Toronto	Canada	7123826	13.21	23.06	33.72
Toulouse	France	1330243	14.27	30.02	42.65
Turin	Italy	1741546	38.38	52.55	61.3
Utrecht	Netherlands	882821	16.21	38.68	54.86
Valencia	Spain	1686890	31.04	56.66	71.73
Vancouver	Canada	2539976	8.52	22.55	34.37
Venice	Italy	557955	13.98	22.21	27.76
Vienna	Austria	2779253	44.63	68.02	78.94
Vilnius	Lithuania	691221	28.55	38.33	43.84
Warsaw	Poland	3099687	26.7	42.78	50.95
Washington	United States	8899517	3.12	8.42	12.89
Winnipeg	Canada	846133	0.0	0.0	0.0
Zurich	Switzerland	1329898	42.7	68.18	82.09
